# Metabolic Adaptation and Potential Regulatory Mechanisms of Longissimus Dorsi-Derived Skeletal Muscle Satellite Cells from Hu Sheep Under Insulin Induction

**DOI:** 10.3390/ani16131954

**Published:** 2026-06-24

**Authors:** Haotian Yuan, Xiongxiong Li, Zengkui Lu, Chao Yuan, Tingting Guo, Lixia Sun, Jianbin Liu, Bowen Chen

**Affiliations:** 1 Key Laboratory of Animal Genetics and Breeding on the Tibetan Plateau, Ministry of Agriculture and Rural Affairs, Lanzhou Institute of Husbandry and Pharmaceutical Sciences, Chinese Academy of Agricultural Sciences, Lanzhou 730050, China; yhtian0125@163.com (H.Y.); lxx_gsau@163.com (X.L.); luzengkui@caas.cn (Z.L.); yuanchao@caas.cn (C.Y.); guotingting@caas.cn (T.G.); sunlixia202109@163.com (L.S.); 2 Sheep Breeding Engineering Technology Research Center of Chinese Academy of Agricultural Sciences, Lanzhou 730050, China

**Keywords:** Hu sheep, skeletal muscle satellite cells, insulin, bidirectional differentiation potential, whole-transcriptome sequencing

## Abstract

The bidirectional differentiation potential of skeletal muscle satellite cells (SMSCs) enables them to differentiate into myofibers or intramuscular adipocytes, which affects meat quality in livestock. However, how insulin regulates ovine SMSC metabolism remains poorly understood. SMSCs were isolated from the longissimus dorsi muscle of 1-day-old Hu sheep, cultured, identified, and induced to differentiate with insulin. After induction, lipid droplet formation and the number of nuclei per cell were assessed, and samples were collected before adipogenic induction (No_AD) and after adipogenic induction (AD) for qPCR and whole-transcriptome sequencing. Immunofluorescence confirmed cells were positive for PAX7 and DESMIN. Bodipy, Oil Red O, and hematoxylin staining revealed lipid droplets and multinucleated cells. Sequencing and qPCR indicated that insulin promoted fatty acid uptake and utilization, inhibited adipogenic differentiation, and promoted myogenic differentiation. Integrated ceRNA analysis suggested that miR-2447-z and MSTRG.8123.1 may coordinate muscle development and lipid metabolism. In conclusion, under insulin induction, ovine SMSCs may undergo metabolic adaptation through the ceRNA network mediated by miR-2447-z and MSTRG.8123.1, exhibiting enhanced myogenesis, suppressed adipogenesis, and lipid droplet accumulation. These findings provide new insights into insulin-regulated SMSC metabolism, suggesting that leveraging the bidirectional differentiation potential of SMSCs to in-fluence muscle characteristics and fat deposition may be a feasible approach for im-proving meat production traits in sheep.

## Simple Summary

Sheep muscle contains specialized cells called satellite cells. These cells have the unique ability to differentiate into either muscle fibers or adipocytes (fat cells). This ability directly affects meat marbling, the intramuscular fat distribution that determines meat tenderness and flavor. However, how the hormone insulin regulates this process remains incompletely understood. In this study, satellite cells were isolated from the longissimus dorsi muscle of Hu lambs, cultivated and treated with insulin. Following insulin treatment, the cells accumulated small lipid droplets and formed multinucleated myotubes, indicating successful muscle fiber development. Comprehensive transcriptomic analysis and quantitative real-time polymerase chain reaction revealed that insulin promoted fatty acid utilization, suppressed adipogenic differentiation, and enhanced myogenic differentiation. Further integrated analysis of competitive endogenous RNA networks indicated that two key molecules, designated miR-2447-z and MSTRG.8123.1, may coordinately regulate muscle fiber development and lipid metabolism. Collectively, these findings demonstrate that insulin induces metabolic adaptation in ovine satellite cells, prioritizing myogenesis over adipogenesis while permitting lipid droplet accumulation. This study provides novel insights into the mechanisms by which insulin regulates satellite cell metabolism and identifies potential molecular targets for improving sheep meat production traits.

## 1. Introduction

Meat quality is of paramount importance in livestock production. The amount of fat deposition and fatty acid composition are key factors influencing meat tenderness, juiciness, and flavor [1,2], while the contractile type, metabolic characteristics, size, and number of muscle fibers also play important roles in determining meat quality [3]. Therefore, both fat and muscle represent important entry points for improving meat quality. Although regulating either one alone is a feasible approach, simultaneously modulating both may yield greater value.

Skeletal muscle satellite cells (SMSCs) are adult stem cells located between muscle fibers and the basement membrane, playing critical roles in skeletal muscle development and regeneration [4]. Their behavior and fate are regulated by both cell-intrinsic factors and external stimuli [5]. Notably, SMSCs possess bidirectional differentiation potential toward myogenic and adipogenic lineages [6,7,8,9], a unique characteristic that makes them ideal target cells for simultaneously regulating muscle growth and fat deposition. The proliferation and differentiation capacity of SMSCs has been utilized by researchers to increase meat production [10] and develop cultured meat [11,12,13,14].

However, SMSCs are committed to myogenic differentiation and do not spontaneously adopt an adipogenic fate [15]. Therefore, regulating the adipogenic differentiation of SMSCs represents a key entry point for leveraging their bidirectional differentiation potential to improve meat quality. Inducing agents such as ciglitazone, adiponectin, and oleic acid have been shown to promote adipogenic differentiation of SMSCs [9,16]. However, it has been reported that, under adipogenic induction conditions, SMSCs accumulate lipids but still maintain myogenic protein expression without fully executing the adipogenic program [15,17]. Insulin is a classic adipogenic inducer commonly used to promote adipogenesis in various cell types [18,19,20]. Under certain conditions, insulin also promotes muscle protein synthesis and inhibits its degradation [21], while also promoting SMSC proliferation and myogenic differentiation [22,23]. Therefore, insulin may exert unique regulatory effects on the bidirectional differentiation fate of SMSCs.

The metabolic response characteristics of SMSCs under induction with insulin remain unclear. Some long non-coding RNAs (lncRNAs) can act as competing endogenous RNAs (ceRNAs) to interact with microRNAs (miRNAs) and regulate messenger RNA (mRNA) expression, thereby influencing the differentiation and development of adipocytes and muscle cells [24,25,26,27]. Therefore, we propose that insulin induces metabolic adaptation in SMSCs through ceRNA-mediated regulatory networks, thereby affecting their myogenic and adipogenic characteristics. In this study, using SMSCs derived from the longissimus dorsi muscle of Hu sheep as a model, we aim to systematically analyze the metabolic response characteristics and potential regulatory networks of SMSCs under induction with insulin through microscopic observation (cell morphology and lipid droplet staining results, etc.) and whole-transcriptome sequencing. The aim is to elucidate the molecular mechanisms by which insulin regulates SMSC metabolism and to provide a theoretical basis for utilizing their bidirectional differentiation potential to improve meat quality.

## 2. Materials and Methods

### 2.1. Sheep Muscle Tissues Collection

This study used three 1-day-old newborn sheep (Hu sheep, 1.5–3 kg, ♂) from Lanzhou Wanshan Plantation and Breeding Professional Cooperative (Lanzhou, China). These animals were anesthetized with inhaled isoflurane (Sigma-Aldrich, St. Louis, MO, USA) and then fixed on a trough-shaped stool. The carotid artery was severed for exsanguination and slaughter. After exsanguination, the skin on the back was incised with a sterile scalpel and the longissimus dorsi muscles between the 12 and 13th rib were dissected. The longissimus dorsi muscles were rinsed with 75% alcohol (Kangzhuangyuan, Dezhou, China) and PBS (Solarbio, Beijing, China), then stored at low temperature in PBS supplemented with 1% penicillin-streptomycin-gentamicin solution (Solarbio, Beijing, China), and transferred to the laboratory. Subsequently, the longissimus dorsi muscles from the three sheep were pooled for cell isolation and culture. The methods for cell isolation, purification, and differentiation induction were adapted from relevant literature, with modifications to meet the requirements of the present study [28,29,30].

### 2.2. Cell Isolation and Culture

The tissue was transferred to a Petri dish and rinsed three times, then transferred to a new Petri dish, where blood vessels, tendons, and fascia were removed and the tissue was cut into a minced consistency with scissors. The minced tissue was mixed with Collagenase II (Solarbio, Beijing, China; 1.0, 1.5, or 2.0 mg/mL) at a ratio of 1:5 (tissue:collagenase) and digested in a 37 °C water bath for 1.5 h, with the centrifuge tube inverted every 5 min to mix. An equal volume of culture medium (containing 90% DMEM/F12, Gibco, Grand Island, NY, USA + 10% FBS, Gibco, Grand Island, NY, USA + 1% Penicillin-Streptomycin-Gentamicin Solution) was added to terminate digestion. The mixture was then passed through 70 μm and 40 μm cell strainers sequentially. The filtered cell suspension was transferred to a 15 mL centrifuge tube, centrifuged (Biofuge Stratos, Thermo Electron Corporation, Osterode, Germany) at 1200 rpm for 10 min, the supernatant was discarded, and the cells were resuspended in complete medium. The cells were seeded into T25 flasks, mixed, and cultured in a CO_2_ incubator (HF90/HT, Heal Force, Shanghai, China) at 37 °C with 5% CO_2_; this passage was designated as passage 0. Cell growth was observed daily, the medium was changed every two days, and the cells were passaged when they reached 80% confluence.

### 2.3. Cell Passage Cultivation

For cell passage, the culture medium was removed, and the cells were washed twice with PBS. After removing the PBS, 0.25% Trypsin-EDTA (Gibco, Grand Island, NY, USA) was added to cover the bottom of the flask. The cells were incubated in the incubator for 2–5 min. Twice the volume of culture medium was added to stop digestion, mixed well, and the cells were detached from the surface by gentle pipetting. The cell suspension was transferred to a 15 mL centrifuge tube, centrifuged at 1200 rpm for 5 min, the supernatant was discarded, and the cells were resuspended in culture medium. The cell suspension was then transferred to a new T25 flask containing complete medium and mixed well. After standing for 2–3 min, cell morphology was observed under a microscope. The flasks were labeled and placed in the incubator.

### 2.4. Cell Purification (Differential Adhesion)

After passaging and 2 h of incubation, the original medium was aspirated, and the cells were washed twice with complete medium. Fresh complete medium was then added, and the flask was returned to the incubator for further culture. This purification procedure was performed twice. After the two purifications were completed, the cells were designated as passage 2. Subsequently, no penicillin-streptomycin-gentamicin solution was added to either the PBS or the complete medium.

### 2.5. Immunofluorescence

Purified cells (passage 3) at 80% confluence were used for immunofluorescence staining. After washing the cells with PBS, they were fixed with 4% paraformaldehyde fixative (BioSharp, Hefei, China) at room temperature for 10 min, then washed three times with PBST (125 µL Tween-20, Bioss, Woburn, MA, USA + 49.875 mL PBS) for 5 min each. The cells were then permeabilized with permeabilization buffer (30 µL Triton X-100, Solarbio, Beijing, China + 9.97 mL D-PBS, BioSharp, Hefei, China) for 10 min, followed by three washes with PBST for 5 min each. Blocking buffer (500 µL FBS + 30 µL Triton X-100 + 9.47 mL PBS) was added to a 6-well plate, and the cells were blocked at room temperature for 1 h. After removing the blocking solution, PAX7 (Cat. No. 20570-1-AP, 1:200 dilution; Proteintech, Rosemont, IL, USA) or Desmin (Cat. No. 16520-1-AP, 1:200 dilution; Proteintech, Rosemont, IL, USA) polyclonal antibody diluted in antibody dilution buffer (19.94 mL D-PBS + 60 µL Triton X-100 + 0.2 g BSA, Sigma-Aldrich, St. Louis, MO, USA) was added to the wells and incubated overnight at 4 °C, followed by three washes with PBST for 5 min each. Cy3-conjugated goat anti-rabbit IgG H+L (Cat. No. SA00009-2, 1:50 dilution; Proteintech, Rosemont, IL, USA) diluted in antibody dilution buffer was then added and incubated for 1 h at room temperature in the dark, followed by three washes with PBST for 5 min each. Finally, 0.4 µg/mL DAPI (Solarbio, Beijing, China) diluted in blocking buffer was added and incubated for 10 min at room temperature in the dark. The cells were then washed three times with PBST for 5 min each, and images were acquired using an inverted fluorescence microscope (CKX53SF, Olympus, Tokyo, Japan) in the dark.

### 2.6. Adipogenic Induction of SMSCs

In the classic adipogenic differentiation cocktail, IBMX and dexamethasone function to initiate the adipogenic program, while insulin plays a dominant role during the execution phase of adipogenesis, indicating that insulin is the primary active substance in this cocktail [20,31,32]. Therefore, this study employed this cocktail to focus on the effects of insulin on the bidirectional differentiation potential of SMSCs. When passage 4 cells reached complete confluence, 16 T25 flasks and 3 six-well plates of cells were used for differentiation induction (after adipogenic induction, AD), and another 16 flasks were cryopreserved for subsequent assays (before adipogenic induction, No_AD). The differentiation procedure was performed as follows: cells were incubated for 2 days in complete medium containing insulin (15 μg/mL, Sigma-Aldrich, St. Louis, MO, USA), dexamethasone (1 μM, MedChemExpress, Monmouth Junction, NJ, USA), and 3-isobutyl-1-methylxanthine (0.5 mM, Sigma-Aldrich, St. Louis, MO, USA). Then, cells were incubated for 2 days in complete medium containing insulin (15 μg/mL) alone. Finally, cells were cultured for an additional 2 days in complete medium.

### 2.7. Bodipy Staining

Six-well plates of AD cells (passage 4) were used for Bodipy staining. The culture medium was removed, and the cells were washed once with PBS. The cells were then fixed with 4% paraformaldehyde at room temperature for 15 min, and the fixative was discarded. Subsequently, 1 μg/mL Bodipy (ChemeGen, Wilmington, MA, USA) staining solution was added, and the cells were incubated at room temperature in the dark for 20 min, and then observed under a microscope.

### 2.8. Oil Red O Staining

Six-well plates of AD cells (passage 4) were used for Oil Red O staining. Staining was performed using the Oil Red O Stain Kit (Solarbio, Beijing, China). The culture medium was removed, and the cells were washed twice with PBS (1 min per wash), then fixed with Oil Red O fixative for 20–30 min. The fixative was discarded, and the cells were washed twice with distilled water, then soaked in 60% isopropyl alcohol (Baishi, Tianjin, China) for 20–30 s. The 60% isopropanol was discarded, and a freshly prepared Oil Red O staining solution was added and incubated for 10–20 min. The staining solution was discarded, and the cells were rinsed with 60% isopropanol for 10–20 s until the stroma was clearly visible. The cells were then washed 2–5 times with distilled water until no excess stain was released. Mayer’s hematoxylin stain was added, and the nuclei were counterstained for 1–2 min. The stain was discarded, and the cells were washed 2–5 times with water. Oil Red O buffer was then added and incubated for 1 min to reverse the blue color, after which it was discarded. Finally, distilled water was added to cover the cells, and images were observed under a microscope.

### 2.9. Whole-Transcriptome Analysis

Six flasks each of AD and No_AD cells (passage 4) were collected (n = 6 per group). The culture medium was removed, and TRIzol (TaKaRa, Shiga, Japan) reagent (1 mL per 10 cm^2^ of culture area) was added. A 1 mL pipette tip was used to pipette up and down repeatedly to ensure thorough contact and lysis of the cells with TRIzol. The solution was then transferred to an RNase-free tube, and a disposable syringe was used to pipette up and down repeatedly until no cell clumps were visible, resulting in a clear, non-viscous solution. The sample was rapidly frozen in liquid nitrogen and then stored at −80 °C. Samples were sent to OmicsMaster Biotechnology Co., Ltd. (Guangzhou, China) for omics (whole-transcriptome) analysis. Detailed sequencing analysis procedures are provided in Appendix A.

### 2.10. Analysis of Gene Expression Related to Cell Metabolism

Four flasks each of AD and No_AD cells (passage 4) were collected for quantitative real-time PCR (qPCR; n = 4 per group). Gene primers were synthesized by Tsingke Biotechnology (Beijing, China), and the primer sequences are listed in Table 1. Total RNA was extracted using the TransZol Up Kit (TransGen, Beijing, China). RNA integrity was verified using agarose gel electrophoresis. RNA concentration was measured using a Qubit 4 Fluorometer (Thermo Fisher Scientific, Waltham, MA, USA). RNA purity requirements: OD 260/280 = 1.9–2.1, OD 260/230 ≥ 1.9. Reverse transcription was performed using the TransScript^®^ One-Step gDNA Removal and cDNA Synthesis SuperMix Kit (TransGen, Beijing, China). PCR amplification was performed using a thermal cycler (ProFlex™ Base, Applied Biosystems, Singapore). The amplification protocol was as follows: incubation at 42 °C for 15 min, followed by 85 °C for 5 s. The reverse-transcribed cDNA was stored at −20 °C until use. qPCR was performed using the TransStart^®^ Top Green qPCR SuperMix Kit (TransGen, Beijing, China). qPCR was performed using a real-time PCR system (LightCycler^®^ 96, Roche, Mannheim, Germany). The amplification protocol was as follows: preincubation at 95 °C for 30 s (1 cycle); 45 cycles of three-step amplification (95 °C for 10 s, 60 °C for 10 s, 72 °C for 20 s); followed by melting curve analysis (95 °C for 10 s, 65 °C for 60 s, 97 °C for 1 s; 1 cycle). Each sample was run in triplicate. β-actin was used as the internal reference gene, and the relative mRNA expression level was calculated using the 2^−ΔΔCt^ method.

### 2.11. Statistical Analysis

Statistical analyses were performed using IBM SPSS Statistics 27. Experimental results were retained to two decimal places, and quantitative data were expressed as mean ± standard error of the mean (Mean ± SEM). Statistical significance was evaluated using one-way analysis of variance (ANOVA), followed by Duncan’s multiple range test. Differences were considered statistically significant at *p* < 0.05.

## 3. Results

### 3.1. Isolation, Culture, and Identification of SMSCs

**Figure 1:**
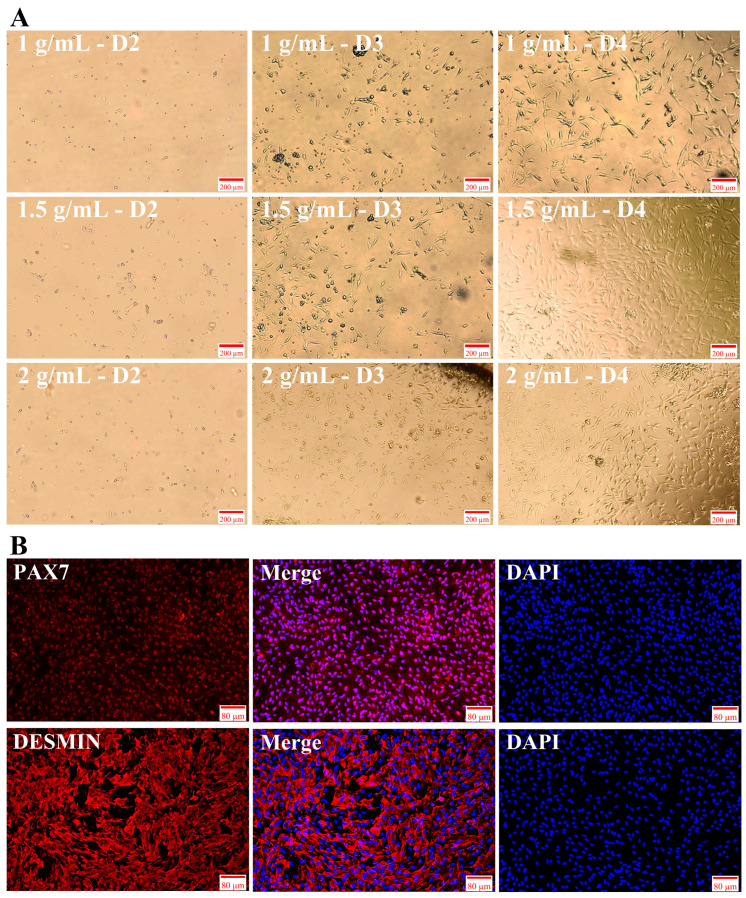
Morphological characteristics and marker identification of isolated SMSCs. (**A**) Morphological characteristics of isolated cells from day 2 to day 4 under different concentrations of Collagenase II. (**B**) Immunofluorescence staining of cell-specific markers. Note: PAX7 and DESMIN are shown in red fluorescence, and DAPI is shown in blue.

Newly isolated SMSCs appeared transparent and round. On the second day of culture, the cells had adhered to the plate, with some cells appearing round and others appearing as short, spindle-shaped or irregular small triangles. Among the treatment groups, the 1.0 mg/mL Collagenase II group had the highest number of round cells. On the third day of culture, the cells began to proliferate, and most cells appeared as short, spindle-shaped or irregular small triangles. However, the 1.0 mg/mL group still contained a large number of round cells, and the cell density in the 1.5 and 2.0 mg/mL groups was higher than that in the 1.0 mg/mL group. On the fourth day of culture, the cells proliferated extensively, appearing spindle-shaped or triangular, with a marked increase in cell density. The cell density in the 1.5 and 2.0 mg/mL groups was greater than that in the 1.0 mg/mL group (Figure 1A). Notably, on the fourth day of culture, the 1.5 mg/mL group contained fewer impurities than the 2.0 mg/mL group. Therefore, 1.5 mg/mL Collagenase II is recommended for isolating SMSCs from the longissimus dorsi muscle. After purification of the isolated SMSCs, specific markers PAX7 and DESMIN were detected using corresponding antibodies. Following staining, the cells exhibited abundant red fluorescence, indicating that the isolated cells were SMSCs. Analysis combined with the blue fluorescence from DAPI staining demonstrated that the cell purity was no less than 95% (Figure 1B).

### 3.2. Insulin Induces Differentiation of SMSCs

**Figure 2:**
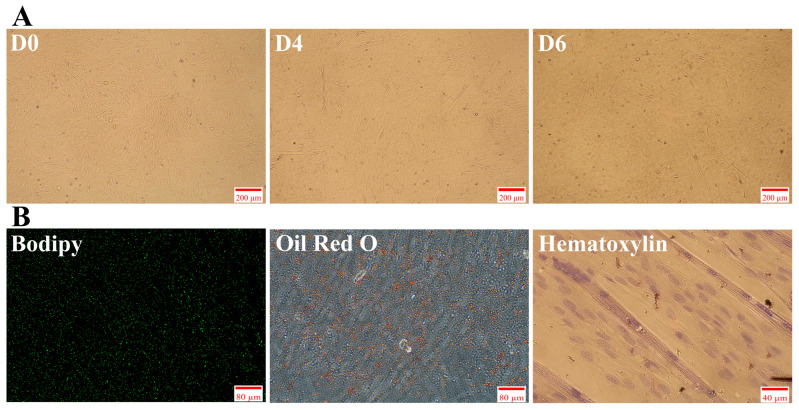
Insulin induces differentiation of SMSCs. (**A**) Morphological characteristics of cells during induction. (**B**) Bodipy, Oil Red O, and hematoxylin staining of cells on day 6 of induction.

When purified SMSCs were cultured until they became a confluent monolayer, this time point was recorded as day 0, and insulin induction was initiated. On day 4 of induction, a large number of small transparent lipid droplets were observed within the cells. On day 6 of induction (day 2 of maintenance culture), the number of lipid droplets further increased (Figure 2A). After insulin induction, the cells were stained to identify lipid droplets. Bodipy staining revealed numerous densely distributed small green fluorescent dots, and Oil Red O staining revealed abundant orange-red lipid droplets (Figure 2B). These results indicated that induction with insulin led to the accumulation of numerous small lipid droplets within the cells. In addition, hematoxylin staining revealed the presence of multinucleated cells, indicating that after insulin-induced differentiation, the cells maintained their myogenic capacity while accumulating a large number of lipid droplets, resulting in the formation of multinucleated myotubes.

### 3.3. Identification of mRNA, lncRNA, and miRNA in SMSCs Before and After Induced Differentiation

**Figure A1:**
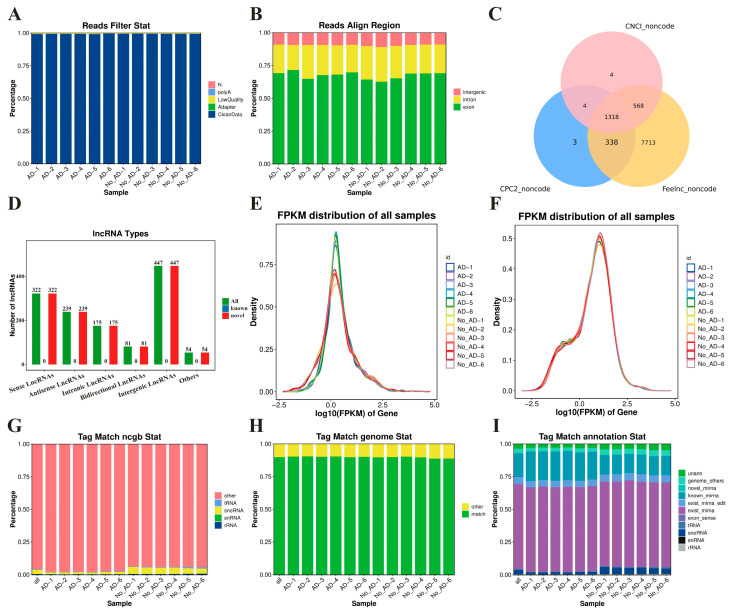
Identification of mRNA, lncRNA, and miRNA in No_AD and AD cells. (**A**) Distribution of data preprocessing. (**B**) Statistical chart of alignment to reference regions. (**C**) Venn diagram of lncRNA prediction. (**D**) Statistical chart of lncRNA types. (**E**) Distribution of lncRNA expression abundance. (**F**) Distribution of mRNA expression. (**G**) Statistical chart of the abundance of tags successfully aligned to non-coding RNAs in ncgb for each sample. (**H**) Statistical chart of the abundance of tags successfully aligned to the genome for each sample. (**I**) Statistical chart of tag abundance for different classifications in each sample.

For mRNA and lncRNA: Clean reads accounted for 99.15–99.59% of the raw reads in each sample (Figure A1A). Among the clean data, the proportion of Q30 bases ranged from 95.33% to 97.43%, and the GC content ranged from 45.19% to 49.97%. After removing ribosomal reads from each sample, the retained unmapped reads accounted for 99.73–99.84% of the clean reads. These reads were then aligned to the reference genome, yielding a total mapped reads alignment rate of 95.89–96.75%. Among the aligned regions, exons accounted for 62.67–71.56% of the total (Figure A1B). A total of 1318 novel lncRNAs were predicted, including 322 sense lncRNAs, 239 antisense lncRNAs, 175 intronic lncRNAs, 81 bidirectional lncRNAs, 447 intergenic lncRNAs, and 54 others (Figure A1C,D). In each sample, 21,525 known mRNAs and 220 novel mRNAs were identified. The distribution of lncRNA and mRNA expression abundance is shown in Figure A1E and Figure A1F, respectively.

For miRNA: Tag represented small RNA sequences. Among the clean reads obtained from each sample, high-quality reads accounted for 99.44–99.79%, and among these high-quality reads, clean tags accounted for 92.53–96.73%. Subsequent alignment to the GenBank and Rfam databases showed that non-coding RNA tag (other) accounted for 93.57–97.89% (Figure A1G). Tag successfully aligned to the reference genome accounted for 88.72–90.40% (Figure A1H), with exons accounting for 0.05–0.19% of the successfully aligned tag. Tags identified as existing miRNAs (miRNAs of this species already annotated in miRBase) accounted for 64.24–66.31% of the total tag abundance. Tag identified as known miRNAs (miRNAs identified by alignment to known animal miRNAs in miRBase) accounted for 14.31–22.54% of the total tag abundance. In addition, novel miRNA tag accounted for 0.10–0.13% of the total tag abundance. The classification annotation composition of tag for each sample was detailed in Figure A1I.

### 3.4. Analysis of Sample Relationships and Differential Expression of mRNA, lncRNA, and miRNA in SMSCs Before and After Induced Differentiation

**Figure 3:**
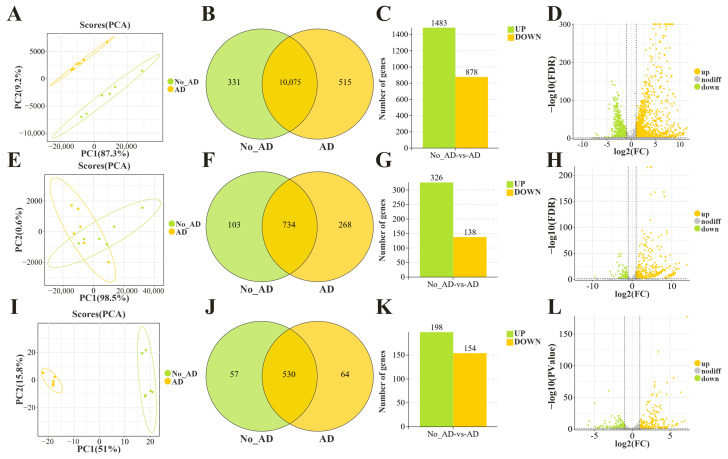
Sample relationships and differential expression analysis of mRNA, lncRNA, and miRNA between No_AD and AD cells. (**A**–**D**) PCA plot of mRNA samples, along with Venn diagram, bar plot, and volcano plot of differentially expressed mRNA. (**E**–**H**) PCA plot of lncRNA samples, along with Venn diagram, bar plot, and volcano plot of differentially expressed lncRNA. (**I**–**L**) PCA plot of miRNA samples, along with Venn diagram, bar plot, and volcano plot of differentially expressed miRNA.

For mRNA, PCA showed complete separation and clear clustering of samples between the two treatments, indicating that insulin treatment had a significant and stable effect on cellular mRNA expression (Figure 3A). The Venn diagram showed 331 unique mRNAs in the No_AD cells, 515 unique mRNAs in the AD cells, and 10,075 shared mRNAs between the two cell types (Figure 3B). The differential bar plot indicated that, compared with the No_AD cells, the AD cells had 1483 significantly upregulated mRNAs and 878 significantly downregulated mRNAs (Figure 3C).

For lncRNA, PCA showed a trend of separation between treatments but with some overlap, suggesting that insulin induction could affect cellular lncRNA expression, but did not completely distinguish it from that of uninduced cells (Figure 3E). The Venn diagram showed 103 unique lncRNAs in the No_AD cells, 268 unique lncRNAs in the AD cells, and 734 shared lncRNAs between the two (Figure 3F). The differential bar plot indicated that, compared with the No_AD cells, the AD cells had 326 upregulated lncRNAs and 138 downregulated lncRNAs (Figure 3G).

For miRNA, PCA showed independent clustering of samples within each treatment and significant separation between treatments, indicating that insulin treatment significantly affected cellular miRNA expression (Figure 3I). The Venn diagram showed 57 unique miRNAs in the No_AD cells, 64 unique miRNAs in the AD cells, and 530 shared miRNAs between the two (Figure 3J). The differential bar plot indicated that, compared with the No_AD cells, the AD cells had 198 upregulated miRNAs and 154 downregulated miRNAs (Figure 3K). In addition, the expression differences in mRNAs (Figure 3D), lncRNAs (Figure 3H), and miRNAs (Figure 3L) between the No_AD and AD cells were reflected in the volcano plots.

### 3.5. Enrichment Analysis of miRNA and ceRNA in SMSCs Before and After Induced Differentiation

**Figure 4:**
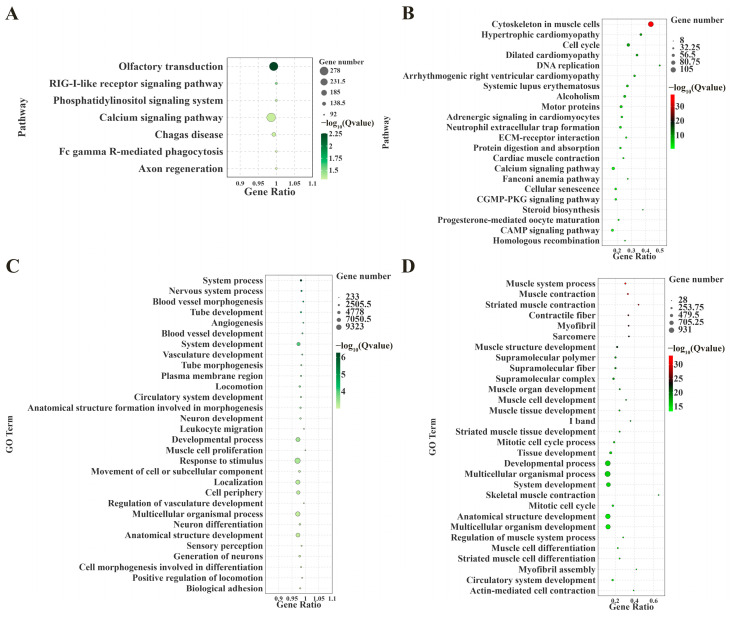
Bubble plots of enrichment analysis of differentially expressed miRNA and ceRNA. (**A**,**B**) KEGG enrichment analysis of miRNA and ceRNA. (**C**,**D**) GO enrichment analysis of miRNA and ceRNA. Note: The screening criterion for miRNA enrichment analysis was *p* < 0.05, and the screening criterion for ceRNA enrichment analysis was FDR < 0.05.

In the KEGG enrichment analysis, the most significantly enriched pathway for miRNA was olfactory transduction, followed by the RIG-I-like receptor signaling pathway. The pathways with the most differentially expressed genes were the calcium signaling pathway (278 genes) and olfactory transduction (272 genes, Figure 4A). For ceRNA, the most significantly enriched pathway was cytoskeleton in muscle cells, followed by hypertrophic cardiomyopathy. The pathways with the most differentially expressed genes were cytoskeleton in muscle cells (105 genes) and cell cycle (51 genes, Figure 4B).

Among the top 30 GO enrichment terms, the most significantly enriched term for miRNA was system process, followed by nervous system process. The terms with the most differentially expressed genes were response to stimulus (9323 genes) and multicellular organismal process (7423 genes, Figure 4C). For ceRNA, the most significantly enriched term was muscle system process, followed by muscle contraction. The terms with the most differentially expressed genes were multicellular organismal process (931 genes) and developmental process (870 genes, Figure 4D).

These results suggest that during induced differentiation, miRNA is involved in regulating calcium-dependent signaling events, immune-related processes, broad cellular signaling responses, and systemic regulation. Meanwhile, ceRNA is closely associated with myofiber formation, acquisition of contractile function, cytoskeletal remodeling, cell cycle exit, and tissue development during skeletal muscle cell differentiation.

### 3.6. Enrichment Analysis of Differentially Expressed mRNA and Screening of Related Genes in SMSCs Before and After Induced Differentiation

**Figure 5:**
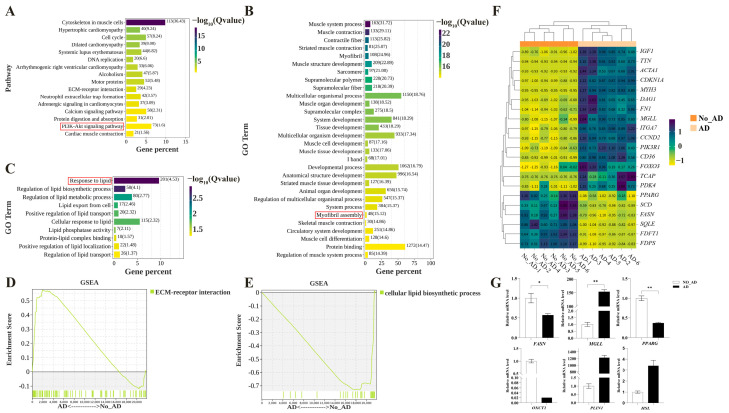
Enrichment analysis of differentially expressed mRNA. (**A**) KEGG enrichment analysis. (**B**) GO enrichment analysis (top 30). (**C**) GO enrichment analysis (lipid metabolism-related). (**D**) KEGG-GSEA analysis. (**E**) GO-GSEA analysis. (**F**) Heatmap of differentially expressed mRNA related to five pathways. (**G**) Bar plot of qPCR validation of mRNA. Note: *p* < 0.05 is marked as “*”, *p* < 0.01 is marked as “**”, and no mark indicates not significant.

In the KEGG enrichment analysis, the most significantly enriched pathway for mRNA was cytoskeleton in muscle cells, followed by hypertrophic cardiomyopathy. The pathways with the most differentially expressed genes were cytoskeleton in muscle cells (113 genes) and the PI3K-Akt signaling pathway (73 genes, Figure 5A). Among the top 30 GO enrichment terms, the most significantly enriched term for mRNA was muscle system process, followed by muscle contraction. The terms with the most differentially expressed genes were protein binding (1272 genes) and multicellular organismal process (1150 genes, Figure 5B). Among lipid metabolism-related terms, the most significantly enriched term for mRNA was response to lipid, followed by regulation of lipid biosynthetic process. The terms with the most differentially expressed genes were response to lipid (201 genes) and cellular response to lipid (115 genes, Figure 5C). These results suggest that during differentiation, mRNA is involved in cytoskeletal remodeling, signal regulation of cell proliferation and differentiation, activation of molecular pathways similar to those in cardiomyopathy, muscle contraction-related processes, protein binding function, and multicellular organismal processes. Meanwhile, insulin induction affects cellular lipid metabolism by regulating pathways related to lipid response and lipid synthesis.

KEGG, GO, and GSEA enrichment analyses all revealed a large number of differentially expressed pathways between the No_AD and AD cells (FDR < 0.05). To systematically dissect the effects of insulin induction on cell differentiation, we focused on five pathways that may play important roles in insulin induction: the PI3K-Akt signaling pathway (signal input, Figure 5A), ECM-receptor interaction (microenvironment perception, Figure 5D), myofibril assembly (identity maintenance, Figure 5B), response to lipid (lipid response, Figure 5C), and cellular lipid biosynthetic process (lipid execution, Figure 5E). We summarized the expression trends of key mRNA involved in these five pathways (Figure 5F), as follows: Compared with the No_AD cells, the AD cells showed upregulation of *IGF1*, *PIK3R1*, *FOXO3*, *CCND2*, and *CDKN1A* in the *PI3K-Akt* signaling pathway; upregulation of *ITGA7*, *DAG1*, *FN1*, and *CD36* in ECM-receptor interaction; upregulation of *MYH3*, *ACTA1*, *TCAP*, and *TTN* in myofibril assembly; upregulation of *CD36* and *PDK4*, and downregulation of *PPARG* and *SCD* in response to lipid; and downregulation of *SQLE*, *FASN*, *FDFT1*, and *FDPS* in cellular lipid biosynthetic process. In addition, qPCR results showed that the relative expression levels of *PPARG* and *FASN* were significantly decreased in the AD cells, while that of *MGLL* was significantly increased in the AD cells (Figure 5G). Their expression trends, along with those of *OXCT1*, *PLIN1*, and *HSL*, were consistent with the sequencing results, indicating that the sequencing data were reliable.

### 3.7. Screening of lncRNA-miRNA-mRNA Interactions

**Figure 6:**
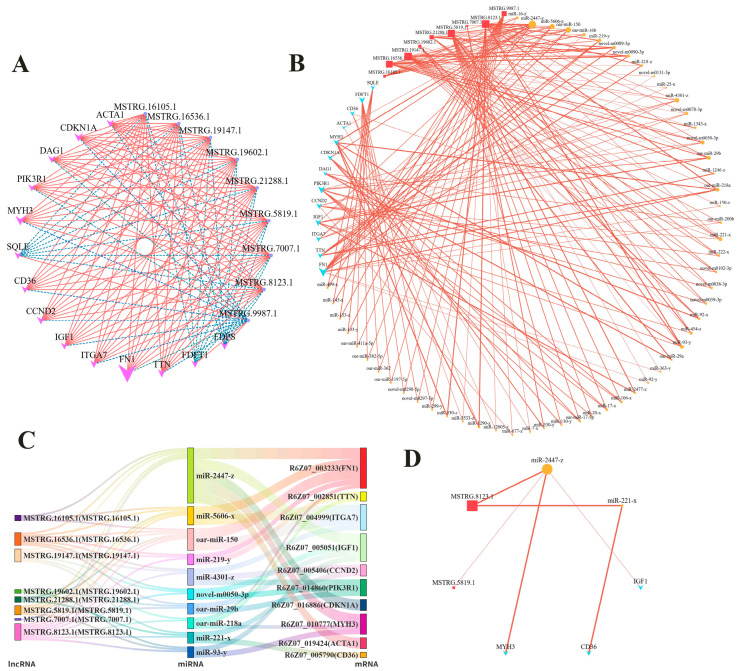
Screening of ceRNA pairs. (**A**) Network plot of trans relationship analysis between lncRNA and mRNA. (**B**) Network plot of lncRNA-miRNA-mRNA interactions. (**C**) Sankey diagram of lncRNA-miRNA-mRNA interactions. (**D**) Final network plot of lncRNA-miRNA-mRNA interactions.

Using trans relationship analysis, we found that 18 mRNA (excluding *PPARG* and *PDK4*) from the five pathways were associated with 185 lncRNA, yielding 1199 lncRNA-mRNA pairs (Table S1). Among these, 9 lncRNAs (MSTRG.16105.1, MSTRG.16536.1, MSTRG.19147.1, MSTRG.19602.1, MSTRG.21288.1, MSTRG.5819.1, MSTRG.7007.1, MSTRG.8123.1, and MSTRG.9987.1) were associated with the highest number of mRNA (14 each), forming 126 pairs (Figure 6A). Based on the lncRNA-mRNA pairs, 124 ceRNA pairs involving 9 lncRNAs and 13 mRNA were identified (Figure 6B, Table S2). By scoring miRNAs (calculated as the product of the number of linked lncRNA and mRNA, sorted from high to low, and taking the top 10), ceRNA pairs associated with 10 key miRNAs were selected (Figure 6C, Table S3).

To screen the core ceRNA regulatory network, we further analyzed all ceRNA pairs involving the highest-scoring miRNA (miR-2447-z). Sorted by lncRNA-mRNA correlation coefficient, the pairs with the highest correlation coefficients were MSTRG.5819.1-miR-2447-z-R6Z07_005051 (r = 0.9915, *p* < 0.01), which is involved in the PI3K-Akt signaling pathway, and MSTRG.8123.1-miR-2447-z-R6Z07_010777 (r = 0.9933, *p* < 0.001), which is involved in myofibril assembly. In addition, *CD36* was the only mRNA among the screened mRNA that was associated with lipid metabolism and linked to two differential pathways. Further analysis revealed a significant correlation for the MSTRG.8123.1-miR-221-x-R6Z07_005790 axis (r = 0.9757, *p* < 0.05), which is involved in the response to lipid pathway. Collectively, we identified three high-confidence ceRNA axes (Figure 6D).

## 4. Discussion

The bidirectional differentiation potential of SMSCs toward myogenic and adipogenic lineages is of great significance for improving meat quality in animal husbandry. In this study, using ovine SMSCs as a model, we aimed to investigate the effects of insulin on their differentiation and the potential regulatory mechanisms involved. The following discussion focuses on phenotypic changes, molecular characteristics, and potential regulatory networks.

At the phenotypic level, this study found cells were positive for PAX7 and DESMIN [6], and multinucleated cells were observed after insulin induction, indicating that the cells still possess myogenic characteristics and have differentiated into multinucleated myotubes [33,34]. In addition, a large number of lipid droplets were produced in the cells after insulin induction.

At the molecular level, differential enrichment analysis indicated that miRNAs may be involved in more upstream or broader regulatory networks (e.g., calcium-dependent signaling events, immune-related processes, etc.), while ceRNAs were more directly associated with muscle-specific differentiation events (e.g., myofiber formation, cytoskeletal remodeling, etc.). mRNAs were involved in both muscle-specific processes and signal regulation and molecular pathway activation. In addition, mRNAs were also involved in the regulation of cellular lipid metabolism by insulin through pathways such as response to lipid, cellular response to lipid, and cellular lipid biosynthetic process. Therefore, we selected five pathways that were significantly enriched with differentially expressed mRNA. Among these, the PI3K-Akt signaling pathway is a core signal transduction pathway in response to external stimuli [35] and plays a critical role in cellular processes such as proliferation, apoptosis, and migration [36,37]. The genes *PIK3R1* [38] and *IGF1* [39] in this pathway are both associated with the insulin receptor protein. Their upregulation indicates that the cells sensed and transduced the insulin signal in the induction medium. *FOXO3* functions through the *PI3K-Akt* signaling pathway to initiate myogenic differentiation of satellite cells [40], and its upregulation reflects the successful transmission of the signal to the effector level. In addition, both *CCND2* and *CDKN1A* were upregulated, with *CDKN1A* showing a greater magnitude of upregulation than *CCND2*. *CCND2* promotes cell proliferation [41], whereas *CDKN1A* primarily functions to arrest cell cycle progression [42]. This suggests that the cell cycle may be in a regulatory state where both “pro-proliferation” and “cell cycle inhibition” coexist, with the inhibition signal being dominant. These findings indicate that the cells successfully received the induction signal, while the cell cycle was precisely regulated.

The extracellular matrix (ECM) is an important component of the stem cell microenvironment and participates in regulating cell behavior and fate. Moreover, different types of stem cells possess various molecules that interact with the ECM [43]. Therefore, we further analyzed genes related to ECM-receptor interaction to understand the cells’ perception of their microenvironment. The proteins encoded by *ITGA7* and *DAG1* maintain normal muscle adhesion and function by linking the cytoskeleton to the ECM [44,45]. Their upregulation suggests that cell adhesion to the basement membrane may be enhanced. Fibronectin 1 (FN1) is involved in cell adhesion, migration, and movement in various cell types [46]. The upregulation of *FN1* suggests active ECM remodeling, possibly to enhance adhesion. Notably, CD36 is a multifunctional receptor that is closely associated with both ECM-receptor interaction and the subsequent response to lipid pathway [47], and it plays a critical role in fatty acid uptake [48]. *CD36* may link microenvironmental perception with lipid metabolism. In addition, its upregulation indicates that the cells actively maintain their skeletal muscle satellite cell identity while also responding to insulin induction.

Myofibril assembly is a direct manifestation of changes in muscle cells. The *MYH3* gene regulates both myofiber type specification and adipogenesis in skeletal muscle [49], and its disruption leads to slowed or even arrested myotube differentiation [50]. The *ACTA1* gene encodes skeletal muscle α-actin, which is the core component of the thin filament of the sarcomere [51]. TCAP is a Z-disc protein that interacts with TTN and participates in the regulation and development of normal sarcomere structure [52]. TTN is a sarcomeric protein involved in constituting the myofibril skeleton [53]. The upregulation of *MYH3*, *ACTA1*, *TCAP*, and *TTN* indicates that the cells are actively synthesizing myofibril structural proteins, demonstrating that insulin induction did not cause the satellite cells to lose their muscle cell identity.

In terms of lipid metabolism, response to lipid reflects the manner in which cells respond to lipid-derived signals in the environment. *PDK4* promotes fatty acid oxidation [54] and inhibits both lipid synthesis [55] and glucose oxidation [56]. The upregulation of both *CD36* and *PDK4* collectively points toward enhanced fatty acid utilization. *PPARG* is a core regulator of lipid metabolism [57] and plays a critical role in adipogenesis and the expression of adipocyte genes [58]. *SCD* is a key rate-limiting enzyme in the synthesis of monounsaturated fatty acids [59]. The downregulation of both *PPARG* and *SCD* clearly indicates that the cells did not initiate an adipogenic differentiation program. Furthermore, cellular lipid biosynthetic process directly addresses whether cells are synthesizing lipids. Fatty acid synthase (FASN) is a key enzyme in de novo fatty acid synthesis, while monoacylglycerol lipase (MGLL) is an important metabolic enzyme that converts triglycerides into free fatty acids [60]. *SQLE*, *FDFT1*, and *FDPS* are all associated with key enzymes involved in cholesterol synthesis [61,62,63]. The downregulation of FASN indicates that de novo fatty acid synthesis was inhibited. The coordinated downregulation of *SQLE*, *FDFT1*, and *FDPS* further indicates that cholesterol synthesis was also suppressed. These gene expression patterns are consistent with the downregulation of *SCD* in the response to lipid pathway, collectively pointing toward the shutdown of lipid synthesis. In parallel, qPCR results showed a significant upregulation of *MGLL*, a key enzyme gene in lipolysis, contrasting with the downregulation of lipid synthesis-related genes. Together, these findings suggest that the cells exhibited a metabolic characteristic of “enhanced breakdown and reduced synthesis” in lipid metabolism. This metabolic pattern may direct fatty acids toward oxidative energy production, thereby providing energy support for active muscle synthesis.

In this study, we found that insulin promoted myogenic differentiation of satellite cells while inducing lipid droplet accumulation, but did not initiate an adipogenic differentiation program. This metabolic characteristic is similar to the findings that quercetin inhibits adipogenic differentiation of muscle satellite cells [64] and that insulin promotes myogenic differentiation of SMSCs [22,23]. To explore the potential regulatory mechanisms underlying the aforementioned expression changes, we performed a series of screenings of differentially expressed genes within the pathways and integrated ceRNA analysis, leading to the identification of three high-confidence axes: MSTRG.5819.1-miR-2447-z-*IGF1* (signal input), MSTRG.8123.1-miR-2447-z-*MYH3* (muscle synthesis), and MSTRG.8123.1-miR-221-x-*CD36* (lipid metabolism). Together, these axes may form a network with miR-2447-z at its core, regulating signal input and muscle synthesis through two distinct lncRNAs. With MSTRG.8123.1 serving as a cross-domain factor, it connects muscle and lipid metabolism via different miRNAs. These two components may together constitute a potential regulatory chain of “signal → muscle → lipid metabolism,” playing a critical role in the metabolic adaptation and identity maintenance of SMSCs following insulin induction.

## 5. Conclusions

In summary, after induction with insulin, SMSCs maintained their myogenic characteristics and did not fully execute the adipogenic program, but accumulated lipid droplets. This suggests that although insulin did not fully drive SMSC adipogenic differentiation, it may still influence meat quality through the regulation of both muscle and fat deposition. Furthermore, the metabolic changes induced by insulin in SMSCs are mediated by three lncRNA-miRNA-mRNA regulatory axes (MSTRG.5819.1-miR-2447-z-*IGF1*, MSTRG.8123.1-miR-2447-z-*MYH3*, and MSTRG.8123.1-miR-221-x-*CD36*), in which miR-2447-z and MSTRG.8123.1 serve as key regulatory molecules coordinating myogenesis and adipogenesis in SMSCs under induction with insulin. This study reveals the metabolic adaptation of SMSCs under induction with insulin, suggesting that their bidirectional differentiation potential may be harnessed to improve meat quality in animal husbandry, and provides potential molecular targets for this purpose. However, the findings of this study are based on in vitro experiments, and the regulatory functions of the identified ceRNA axes have not been experimentally validated. Future studies incorporating functional validation and in vivo research are needed to confirm these findings.

## Figures and Tables

**Table 1:** RT-qPCR primers.

Gene	Primer Sequence (5′–3′)		bp	GenBank
*PPARG*	Forward	ATAAAGCGTCAGGGTTCCAC	115	NM_001100921.1
	Reverse	ATCCGACAGTTAAGATCACACC		
*FASN*	Forward	AAGTTAAAGAATTTGTTCGGA	132	AB011671.1
	Reverse	TCTTCCCGTGACTTTGGTA		
*MGLL*	Forward	GGCGACACTTTTCAAGGTCTTC	132	XM_012100295.4
	Reverse	AGGGGGTCCGAGTTGTAGAT		
*OXCT1*	Forward	TTCCTTTTCCTCCCGTCCAG	182	XM_004017014.6
	Reverse	GTAGCAAATACATCCCTTGGACC		
*PLIN1*	Forward	GGCTGTCCACCCAGTTTGTAG	163	NM_001113773.1
	Reverse	ACACTGATGCTGTTCCTGGC		
*HSL*	Forward	AGTACGTCACGCTGCACAAA	157	NM_001128154.1
	Reverse	CTGGCGGTCACACCGAT		
*β-actin*	Forward	CGAGCACGATGAAGATCAAGATTA	186	XM_004013078.5
	Reverse	TACGCATCTGCTCGCAGTC		

## Data Availability

The data presented in this study are deposited in the SRA repository, accession number: PRJNA1459064. Our data have been released (https://www.ncbi.nlm.nih.gov/bioproject/PRJNA1459064, accessed on 23 June 2025).

## Supplementary Materials

The following supporting information can be downloaded at: https://www.mdpi.com/article/10.3390/ani16131954/s1, Table S1: lncRNA-mRNA pairs; Table S2: ceRNA pairs; Table S3: The finally screened ceRNA pairs.

## Abbreviations

The following abbreviations are used in this manuscript:

SMSCs	Skeletal muscle satellite cells
ceRNA	Competitive endogenous RNA
lncRNA	Long non-coding RNA
miRNA	microRNA
mRNA	Messenger RNA
rRNA	Ribosome RNA
FPKM	Fragment per kilobase of transcript per million mapped reads
KEGG	Kyoto Encyclopedia of Genes and Genomes
GO	Gene Ontology
TPM	transcripts per million
PCA	Principal component analysis
FDR	false discovery rate
qPCR	Quantitative real-time PCR
SEM	standard error of the mean
ANOVA	One-way analysis of variance
ECM	extracellular matrix
FN1	Fibronectin 1
FASN	Fatty acid synthase
MGLL	Monoacylglycerol lipase

## Appendix A

### Appendix A.1. Construction of mRNA, lncRNA and miRNA Libraries and Sequencing

Total RNA was extracted from cells using TRIzol reagent. RNA integrity and DNA contamination were assessed by agarose gel electrophoresis. RNA purity was detected using a Nanodrop spectrophotometer (Thermo Fisher Scientific), and RNA integrity was further accurately assessed using an Agilent 2100 (Agilent Technologies, Santa Clara, CA, USA). Libraries were constructed for messenger RNA (mRNA), lncRNA, and miRNA, respectively.

For mRNA and lncRNA: rRNA was removed. For mRNA, mRNA Capture Beads were used to enrich and purify mRNA, followed by fragmentation using high temperature (for lncRNA, fragmentation was performed directly after rRNA removal). The fragmented mRNA was used as a template to synthesize first-strand and second-strand cDNA sequentially. Purified cDNA was subjected to end repair, A-tailing, and adapter ligation (for lncRNA, the second strand was degraded by Uracil-N-Glycosylase). AMPure XP beads were used to select cDNA fragments of approximately 200 bp, followed by PCR amplification and re-purification to obtain the final library (for lncRNA, agarose gel electrophoresis was used for cDNA size selection before PCR amplification).

For miRNA: Total RNA fragments of 18–30 nt were selected and recovered by agarose gel electrophoresis. 3′ and 5′ adapters were ligated sequentially, and the small RNA with both adapters was reverse transcribed and PCR amplified. Finally, agarose gel electrophoresis was used to recover and purify the ~140 bp band to complete library construction. All libraries were quality-checked and sequenced using the Illumina Novaseq X Plus.

### Appendix A.2. Primary Analysis

For mRNA: To obtain high-quality clean reads, reads were further filtered using fastp (version 0.18.0) [65] with the following parameters: (1) reads containing adapters were removed; (2) reads containing more than 10% unknown nucleotides (N) were removed; (3) low-quality reads containing more than 50% low-quality (Q-value ≤ 20) bases were removed. Bowtie2 (version 2.2.8) [66] was used to map reads to the ribosomal RNA (rRNA) database, and rRNA-mapped reads were removed. The remaining clean reads were used for assembly and gene abundance calculation. An index of the reference genome was built, and paired-end clean reads were mapped to the reference genome using HISAT2 (version 2.1.0) [67] with default parameters. The mapped reads of each sample were assembled using StringTie (version v1.3.1) [68,69,70] in a reference-based approach. For each transcription region, the FPKM (fragments per kilobase of transcript per million mapped reads) value was calculated using RSEM [71] software (version 1.2.19) to quantify expression abundance and variations.

For lncRNA: Unlike mRNA, the processing of lncRNA did not end after the assembly and reconstruction of transcripts to identify new genes and new splice variants of known ones. Transcripts were classified using Cuffcompare, and transcripts with one of the class codes “u, i, j, x, c, e, o” were defined as novel transcripts. Among these, transcripts with a length longer than 200 bp and more than 2 exons were considered reliable novel genes. Novel transcripts were then aligned to the Nr, Kyoto Encyclopedia of Genes and Genomes (KEGG), and Gene Ontology (GO) databases to obtain protein functional annotation. Three software tools—CNCI (version 2) [72], CPC (version 0.9-r2) [73], and FEELNC (version v0.2) [74] were used to assess the protein-coding potential of novel transcripts. The intersection of non-protein-coding potential results from all three tools was selected as long non-coding RNAs. The mapped reads of each sample were assembled using StringTie v1.3.1 in a reference-based approach. For each transcription region, the FPKM value was calculated using RSEM software [71] to quantify expression abundance and variations.

For miRNA: To obtain clean tags, raw reads were further filtered according to the following rules: (1) low-quality reads containing more than one low-quality (Q-value ≤ 20) base or containing unknown nucleotides (N) were removed; (2) reads without 3′ adapters were removed; (3) reads containing 5′ adapters were removed; (4) reads containing both 3′ and 5′ adapters but no small RNA fragment between them were removed; (5) reads containing polyA in the small RNA fragment were removed; (6) reads shorter than 18 nt (excluding adapters) were removed. All clean tags were aligned with small RNAs in the GeneBank database (Release 209.0) and Rfam database (Release 11.0) [75] to remove rRNA, scRNA, snoRNA, snRNA, and tRNA; aligned with the reference genome, and tags mapped to exons or introns were removed; tags mapped to repeat sequences were also removed. Clean tags were then searched against the miRBase database (Release 22) to identify known miRNAs. For species not included in miRBase, alignment with miRNAs of other species was used to identify known miRNAs. All unannotated tags were aligned with the reference genome, and novel miRNA candidates were identified based on genome positions and hairpin structures predicted by miRdeep2 [76]. Unannotated tags were recorded as “unann”. Total miRNA consisted of known miRNAs and novel miRNAs. Based on their expression in each sample, miRNA expression levels were calculated and normalized to transcripts per million (TPM).

### Appendix A.3. Functional and Differential Analysis

Principal component analysis (PCA) and Pearson correlation were performed based on sample RNA expression levels to assess experimental reproducibility. Differential expression analysis was conducted using DESeq2 with read counts, and significantly differentially expressed RNAs were screened with false discovery rate (FDR) < 0.05 and |log_2_FC| > 1. GO and KEGG enrichment analyses were performed using the hypergeometric test. Meanwhile, GSEA was conducted by ranking genes according to signal-to-noise ratio, calculating enrichment scores, and performing permutation test correction, ultimately revealing enriched functions and pathways of differentially expressed genes.

### Appendix A.4. ceRNA Analysis

We performed differential expression analysis on various types of RNA based on expression levels. Significantly differentially expressed RNAs were identified as follows: mRNA, lncRNA, and circRNA with FDR < 0.05 and |log_2_FC| > 1, and miRNA with *p* < 0.05 and |log_2_FC| > 1. We then filtered the differentially expressed RNAs to identify potential ceRNAs based on interactions, considering the following three aspects: (1) the targeting relationship between miRNAs and candidate ceRNAs, as well as negative correlations in their expression levels; (2) positive correlations in expression levels among candidate ceRNAs; and (3) the enrichment of candidate ceRNAs that bind to the same miRNA.
